# Chronic treatment with epigallocatechin gallate reduces motor hyperactivity and affects *in vitro* tested intestinal motility of spontaneously hypertensive rats

**DOI:** 10.3402/fnr.v60.28373

**Published:** 2016-02-17

**Authors:** Maria Assunta Potenza, Monica Montagnani, Carmela Nacci, Maria Antonietta De Salvia

**Affiliations:** Department of Biomedical Sciences and Human Oncology, University of Bari ‘Aldo Moro’, Bari, Italy

**Keywords:** green tea, weight gain, colon, duodenum

## Abstract

**Background:**

Green tea catechins seem to contribute toward reducing body weight and fat.

**Objective:**

We aimed to investigate whether chronic administration of (–)-epigallocatechin-3-gallate (EGCG), the most abundant catechin of green tea, reduces weight gain in spontaneously hypertensive rats (SHR), an animal model of metabolic syndrome, by increasing motor activity and/or by altering gastrointestinal motility.

**Design:**

Nine-week-old SHR were randomly assigned to two groups and treated by gavage for 3 weeks with vehicle dimethyl sulfoxide or EGCG (200 mg/kg/day). Age-matched Wistar-Kyoto (WKY) control rats were treated with vehicle alone. The effect of chronic administration of EGCG was evaluated on open-field motor activity and on *ex vivo* colonic and duodenal motility. Moreover, *in vitro* acute effect of 20-min incubation with EGCG (100 µM) or vehicle was evaluated in colonic and duodenal specimens from untreated WKY rats and SHR.

**Results:**

Vehicle-treated SHR were normoglycemic and hyperinsulinemic, and showed a reduction of plasma adiponectin when compared to vehicle-treated WKY rats. In addition, consistent with fasting glucose and insulin values, vehicle-treated SHR were more insulin resistant than age-matched vehicle-treated WKY rats. Chronic treatment for 3 weeks with EGCG improved insulin sensitivity, raised plasma adiponectin levels, and reduced food intake and weight gain in SHR. Vehicle-treated SHR showed increased open-field motor activity (both crossings and rearings) when tested after each week of treatment. The overall hyperactivity of vehicle-treated SHR was significantly reduced to the levels of vehicle-treated WKY rats after 2 and 3 weeks of EGCG treatment. Colonic and duodenal preparations obtained from SHR chronically treated *in vivo* with EGCG showed reduced responses to carbachol (0.05–5 µM) and increased inhibitory response to electrical field stimulation (EFS, 1–10 Hz, 13 V, 1 msec, 10-sec train duration), respectively. *In vitro* acute EGCG incubation (100 µM, 20 min) of colonic and duodenum strips obtained from untreated SHR and WKY rats showed a reduced contractile colonic response to a fixed dose of carbachol (1.5 µM) only in SHR with respect to its own vehicle, whereas the inhibitory duodenal response to a fixed EFS frequency (5 Hz) was significantly reduced in both WKY rats and SHR groups with respect to their own vehicle.

**Conclusions:**

These data suggest that EGCG affects body weight gain in rats and this effect seems to be due to the altered intestinal motility and not to increased motor activity.

Green tea drinking is becoming increasingly a pleasant way to keep the body in good health. Several epidemiological studies support the beneficial effect of green tea consumption, because it lowers rates of certain cancers and is associated with reduced risk of cardiovascular diseases (1, 2). Moreover, antiobesity, antiatherosclerotic, antidiabetic, antibacterial, and antiviral effects have been documented (3–6).

Many of the beneficial effects of green tea are related to its catechin content, particularly (−)-epigallocatechin-3-gallate (EGCG), which is the most abundant catechin of the green tea. Not surprisingly, EGCG can be considered one of the most popular nutraceutical ingredients in the world.

However, several reports suggest that most of the ingested EGCG apparently does not get into the blood. In this regard, a recent evaluation of the biodistribution of EGCG in rats using the positron emission tomography indicated that most of orally administered EGCG was present in the stomach until 60 min after administration. A small amount passed to the blood after intestinal absorption and accumulated in the liver where it was metabolized and returned to the small intestine via the bile (7). Then, significant quantities pass from the small to the large intestine where they are degraded by local microbiota (8).

Studies on healthy individuals have shown that, after oral administration of EGCG, the plasma levels of the catechin are in the range of nanomolar, whereas the studies showing *in vitro* activity are carried out using micromolar EGCG concentrations. Therefore, it is possible to assume that, after oral administration, the gastrointestinal tract may be the area where the EGCG reaches the concentration values used in *in vitro* experiments (9–12).

It is possible to hypothesize that tea drinking can affect the gastrointestinal tract that has direct contact with tea solution and with usually high concentrations of its components because at the gut levels they are absorbed and/or confined and/or recirculated. In fact, it has been shown that in the gastrointestinal tract green tea increases expression of endogenous antioxidants and induces phase I cytochromes P450 and phase II detoxification enzymes so that procarcinogen formation is inhibited with chemopreventive effects on carcinogenesis [see Ref. (13)].

We have previously shown that chronic EGCG oral treatment improved cardiovascular and metabolic pathophysiology in spontaneously hypertensive rats (SHR), a genetic model of hypertension with features of human metabolic syndrome including fasting hyperinsulinemia, insulin resistance, and overweight (14). Moreover, we have shown that chronic EGCG was able to hinder body weight increase in SHR, in agreement with literature data (14).

The present study was undertaken to further investigate whether the lack of body weight gain observed in SHR chronically treated with EGCG could be due to increased energy expenditure and/or to gastrointestinal effect of EGCG. Therefore, we evaluated the motor activity of vehicle-treated Wistar-Kyoto (WKY) rats, vehicle-treated SHR, and SHR treated with EGCG, assessing the responses obtained after 1, 2, and 3 weeks of treatment. At the end of treatment, we evaluated the effects of EGCG on colonic and duodenal activities performing *ex vivo* experiments in specimens obtained from vehicle-treated WKY rats, vehicle-treated SHR, and SHR treated chronically with EGCG *in vivo*. In order to investigate the local effect of EGCG, colonic and duodenal specimens from untreated WKY rats and SHR underwent 20-min incubation with EGCG (100 µM) or vehicle and their responses were examined.

## Materials and Methods

### Experimental animals

All procedures in animals were performed in accordance with Guidelines and Authorization for the Use of Laboratory Animals (Italian Government, Ministry of Health). All experimental procedures were approved by the Committee on the Ethics of Animal Experiments of the University of Bari (protocol number: 60901-X/10; permit number 15/12).

The minimum number of animals was used. All animals were treated humanely according to the institutional guidelines, with due consideration to the alleviation of distress and discomfort.

Nine-week-old male SHR and age-matched normotensive WKY control rats (Harlan, San Pietro al Natisone, Udine, Italy) were used. Rats were housed in an animal facility with monitored temperature and light (12-h cycle and 21±2°C). All rats had free access to water and food. Body weight was measured weekly and food intake was measured daily.

The animals were allowed to acclimate to the environment for at least 7 days. All animals were handled and trained for at least 1 week to reduce the possible stress induced by drug administration. Then, SHR were randomly assigned to two treatment groups and daily treated for 3 weeks by gavage with EGCG (200 mg/kg) or vehicle dimethyl sulfoxide (DMSO, 1 ml/kg). WKY rats were treated daily by gavage for 3 weeks with vehicle alone. Doses of drugs were chosen based on our and literature published studies (14, 15).

During the 3-week investigation, EGCG was well tolerated and acute or chronic toxic effects were not observed in treated animals with respect to vehicle-treated controls. In fact, no significant overt physical or behavioral changes, suggestive of discomfort or toxic effect, were observed during the indicated period.

### Open-field motor activity

Six to eight rats for each experimental group were tested at the end of each week of treatment. However, on the testing day, rats were subjected to evaluation of open-field motor activity just before drug administration in order to avoid the evaluation of acute effect of the drug. The open field area (50 cm×50 cm×50 cm) was made of acrylic with black walls and white floor divided into 16 squares of equal area. Rats were placed in the center of the open field area and left free to explore for 30 min. The observed parameters were the number of squares crossed with forepaws (crossings) and number of rearings (defined as the raising of both forepaws and movement of body to a vertical plane). Motor activity is a combined measure of the number of crossings and rearings. Tests were carried out in a quiet room with background noise, illuminated by a 20-W white light source suspended 2 m above the apparatus. Immediately after each test, the open field was thoroughly cleaned by cotton pads wetted with 96% ethanol solution.

Each session was recorded by videotape and analyzed by a trained scorer, blind with respect to treatment, at a later date.

### Biochemical analysis

After 12-h fasting, blood samples were collected by cardiac puncture from rats anesthetized with sodium pentobarbital (80 mg/kg/ i.p.), heparinized (400 UI/100 g/ i.p.), and sacrificed by cervical dislocation. Serum samples were stored at −80 °C until assay. Then, serum concentrations of insulin (Linco Research, St. Charles, MO, USA) and adiponectin (B-Bridge, Sunnyvale, CA, USA) were measured by ELISA. Plasma glucose concentrations were assessed with a diagnostic glucometer (Accu-Chek Active, Roche Diagnostic, D-68298 Mannheim, Germany). Insulin sensitivity was determined by quantitative insulin sensitivity check index (QUICKI), calculated according to the following equation: 1/[Log (fasting insulin, µU/ml)+Log (fasting glucose, mg/dl)] (16). For QUICKI analysis, triplicate values of six independent measures for fasting glucose and fasting insulin values were obtained from each group of animals.

### Tensiometric studies

Sections of colon and duodenum were isolated and removed after a midline incision of the abdomen. Specimens of duodenum (1 cm in length) or colon (0.5 cm) were immediately placed in a cooled modified Krebs’ solution (pH=7.4) of the following composition (mM): NaCl 113, KCl 4.8, MgSO_4_ 1.2, CaCl_2_(H_2_O) 2.2, NaH_2_PO_4_ 1.2, NaHCO_3_ 25, glucose 5.5, and ascorbic acid 5.5. Specimens were then cleaned, rinsed, and mounted in an organ bath (20 ml) filled with modified Krebs’ solution, maintained at 37°C and aerated with a mixture of 95% O_2_ and 5% CO_2_. One end of the specimen was connected to a metal rod while the other end was attached to a strain gauge transducer (FORT 25, WPI, Sarasota, FL, USA). Isometric tension was measured by the PowerLab data acquisition system and recorded using Chart 5.5.5 (ADInstruments, Castle Hill, Australia). The tissue was allowed to equilibrate for at least 20–30 min prior to the start of the experiment. An initial load of 1.0 g or 0.5 g tension was applied to the duodenum and colon preparations, respectively.

As far as specimens obtained from *in vivo* EGCG-treated SHR, vehicle-treated SHR, and vehicle-treated WKY, colon preparations were tested to evaluate the contractile response to carbachol (0.05–5 µM) and duodenum preparations were tested to evaluate the inhibitory response to transmural stimulation (EFS, 1–10 Hz, 13 V, 1 msec, 10-sec train duration) through two parallel platinum electrodes connected to a stimulator (Digital Stimulator, LE 12106, Letica, Ugo Basile, Italy). In particular, we evaluated the minimum value of basal tone reached during inhibitory response obtained after EFS that was expressed as percentage of the maximal effect elicited by sodium nitroprussiate (SNP, 50 µM). To evaluate the NO-component of the inhibitory response, EFS-induced relaxation was assessed before and after N^G^-nitro-l-arginine (L-NNA, 20 min, 100 µM).

To evaluate *in vitro* acute effect of EGCG, colonic and duodenal specimens from both untreated WKY rats and SHR were tested to measure the contractile response to a fixed concentration of carbachol (1.5 µM) and the inhibitory response to a fixed frequency of EFS (5 Hz, 13 V, 1 msec, 10-sec train duration) before and after 20-min incubation with EGCG (100 µM), respectively.

To obtain a non-adrenergic, non-cholinergic inhibitory response, all experiments with duodenum strips were carried out in the presence of atropine (3 µM) and guanethidine (3 µM) (17).

### Drugs and chemicals

The following drugs were used: EGCG, DMSO, atropine sulfate, guanethidine monosulfate, SNP, L-NNA (Sigma Chemical Co., St. Louis, Missouri, USA). For *in vivo* study, stock solutions of EGCG in DMSO (10%) were prepared and diluted (4× dilution) in drinking water just before intragastric administration. Vehicle-treated WKY rats and SHR received the same amount of DMSO as drug-treated animals.

### Statistical analysis

Statistical analysis was performed by means of one-way or two-way analysis of variance, as appropriate, followed by paired *t*-test with Bonferroni's correction or Newman-Keuls multiple comparison test. All statistical tests were performed with Statsoft software (Release 5). The level of significance was set at *p*<0.05. Results are expressed as the mean±S.E.M. For each experiment six to eight rats or preparations were used.

## Results

### Effect of chronic EGCG treatment on body weight and food intake

**Fig. 1:**
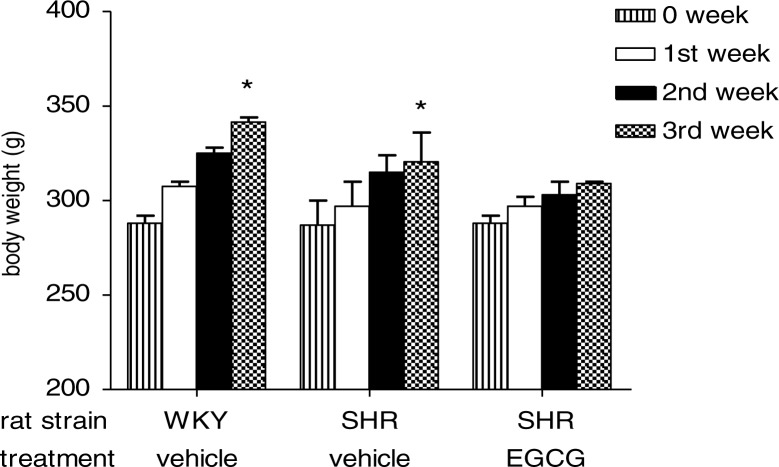
Effect of chronic EGCG treatment on body weight. Values are expressed as mean±SEM of 6–8 rats for each experimental group (*p<0.05 vs week 0 of its own group).

Body weight of vehicle-treated WKY rats and vehicle-treated SHR raised steadily throughout 3 weeks of observation resulting in a significant increase at the end of the third week if compared to body weight recorded at the beginning of treatment (*p< 0.05 vs week 0). Interestingly, the body growth curve of SHR treated with EGCG did not show the same pattern: the rate at which rats gained weight over the 3-week period was slightly slower without reaching a significant increase at the end of EGCG treatment (Fig. 1).

**Fig. 2:**
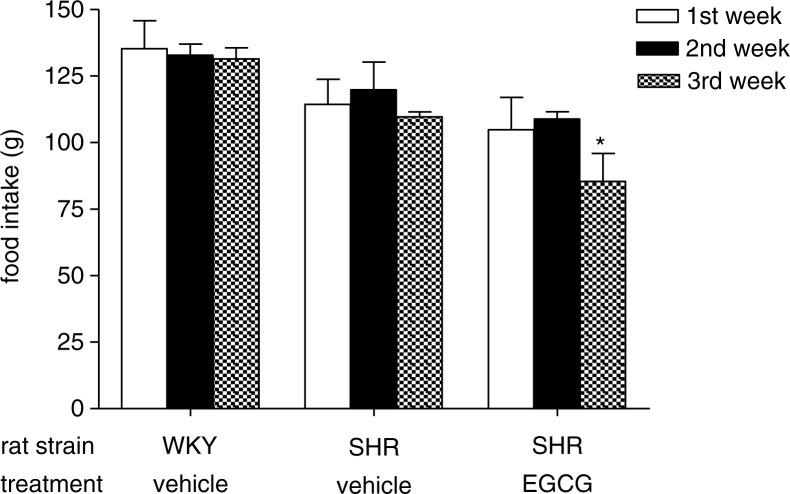
Effect of chronic EGCG treatment on food intake. Values are expressed as mean±SEM of 6–8 rats for each experimental group (*p<0.05 vs vehicle-treated SHR at same time point).

When we evaluated food intake, the results showed no significant difference among the study groups for the first 2 weeks. However, after the third week of treatment, EGCG- treated SHR had a moderate but significant decrease in food intake compared to vehicle-treated SHR at same time point (*p<0.05) (Fig. 2).

These findings are in agreement with our previous research (14).

### Effect of chronic EGCG treatment on open-field motor activity

**Fig. 3:**
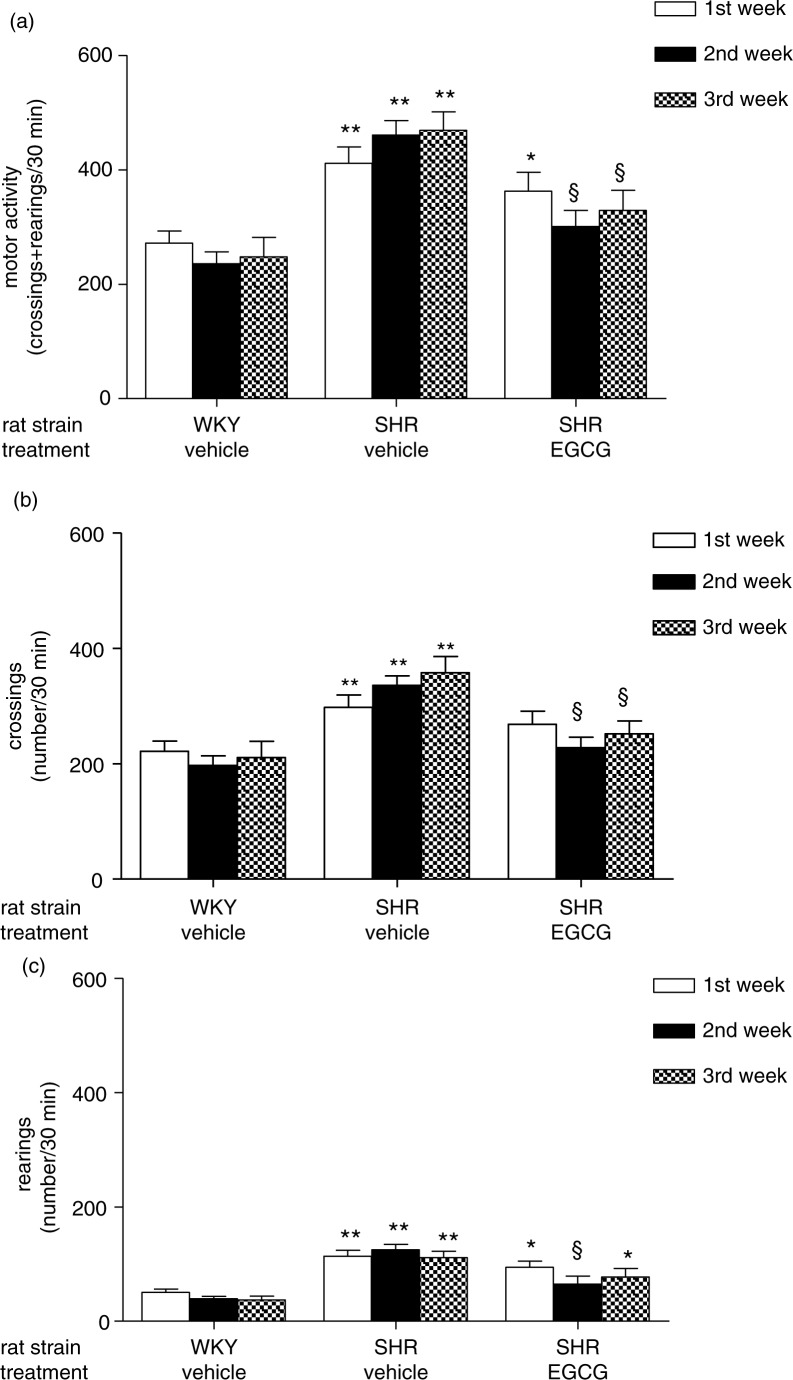
Effect of chronic EGCG treatment on motor activity. (a) Motor activity (i.e. crossings+rearings) recorded in a session of 30 min; (b) number of crossings recorded in a session of 30 min; (c) number of rearings recorded in a session of 30 min. Values are expressed as mean±SEM of 6–8 rats for each experimental group. **p<0.001 and *p<0.05 vs vehicle-treated WKY rats at same time point; ^§^ *p<*0.001 vs vehicle-treated SHR at same time point.

The evaluation of data obtained from open-field motor activity tests showed significantly higher levels of activity in vehicle-treated SHR when compared to vehicle-treated WKY rats at each of three test sessions carried out at the end of each week of treatment (**p<0.001). Interestingly, motor activity of EGCG-treated SHR was significantly higher when compared to vehicle-treated WKY rats (*p<0.05) only after 1 week of treatment. In fact, after 2 and 3 weeks of treatment, EGCG-treated SHR showed motor activity levels comparable to vehicle-treated WKY rats and significantly lower when compared to vehicle-treated SHR at the same time points (§*p*<0.02) (Fig. 3a).

A more in-depth analysis revealed a similar, though not identical, pattern when the number of crossings, one of the two components of motor activity analyzed, was evaluated. In fact a significant increase of number of crossings was found during all periods of study in vehicle-treated SHR (**p<0.001 vs WKY rats). EGCG-treated SHR exhibited a significant decrease of number of crossings reaching control levels and showing a significant difference with respect to vehicle-treated SHR after 2 and 3 weeks of treatment (§*p*<0.01 vs vehicle-treated SHR) (Fig. 3b).

The analysis of the second component of motor activity evaluated, i.e. rearing, showed a significant increase in vehicle-treated SHR during all periods of study (**p<0.001 vs vehicle-treated WKY rats). The number of rearings was not affected by 1 week of treatment with EGCG in SHR and it was significantly higher than vehicle-treated WKY rats at the same time point (*p<0.05). However it was reduced to control levels after 2 weeks of treatment so that was significantly lower with respect to vehicle-treated SHR (§*p*<0.001 vs vehicle-treated SHR). Unfortunately, EGCG-treated SHR showed a significant increase of number of rearings with respect to vehicle-treated WKY after 3 weeks of treatment probably because the reducing effect underwent tolerance (*p<0.05) (Fig. 3c).

### Effect of chronic EGCG treatment on insulin resistance and associated metabolic parameters

**Fig. 4:**
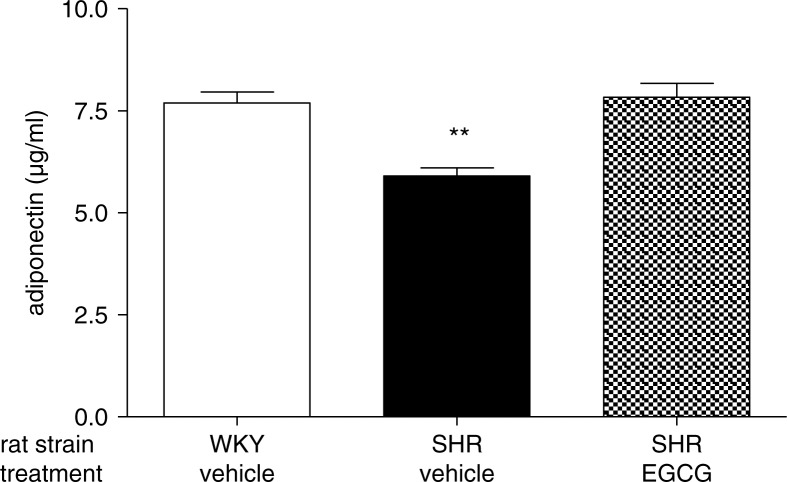
Effect of chronic EGCG treatment on adiponectin. Values are expressed as mean±SEM of 6–8 rats for each experimental group. **p<0.001 vs vehicle-treated WKY rats.

Consistent with our previous investigation (14), plasma levels of adiponectin were found significantly decreased in vehicle-treated SHR when compared to vehicle-treated WKY rats (**p<0.001). SHR chronically treated with EGCG showed restored levels of adiponectin that were not different with respect to the levels of vehicle-treated WKY rats (Fig. 4).

**Fig. 5:**
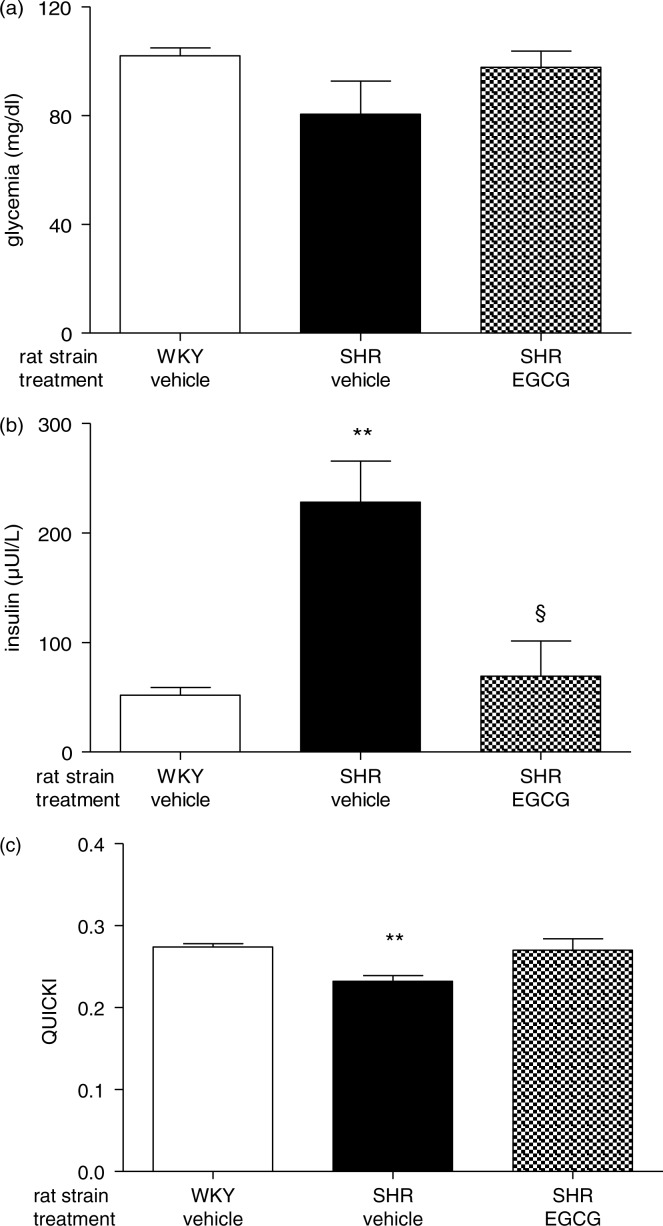
Effect of chronic EGCG treatment on glycemia, insulin levels and QUICKI. (a) Fasting glucose levels; (b) fasting insulin levels; (c) quantitative insulin sensitivity check index (QUICKI). Values are expressed as mean±SEM of 6–8 rats for each experimental group. **p<0.001 vs vehicle-treated WKY rats; ^§^ *p<*0.05 vs vehicle-treated SHR.

Fasting glucose levels were not different between groups but fasting insulin levels were significantly higher in vehicle-treated SHR (**p<0.001 vs vehicle-treated WKY rats). However, SHR treated chronically with EGCG were not hyperinsulinemic and their fasting insulin levels were significantly lower than vehicle-treated SHR (§*p*<0.05). Vehicle-treated SHR were insulin resistant as assessed by QUICKI (16) that was significantly lower than vehicle-treated WKY (**p<0.001). Chronic treatment with EGCG fully restored insulin sensitivity in SHR and QUICKI nearly reached significancy compared to EGCG-treated SHR (*p*=0.07) (Fig. 5a–c).

### Effect of chronic EGCG treatment on *ex vivo* colonic and duodenal motility

To evaluate whether the lack of body weight gain, observed in SHR after 3-week treatment of EGCG, could be due to alteration of gastrointestinal motility, we evaluated the contractile response to increasing concentrations of carbachol (0.05–5 µM) on isolated *ex vivo* colonic specimens obtained from all groups of animals studied.

**Fig. 6:**
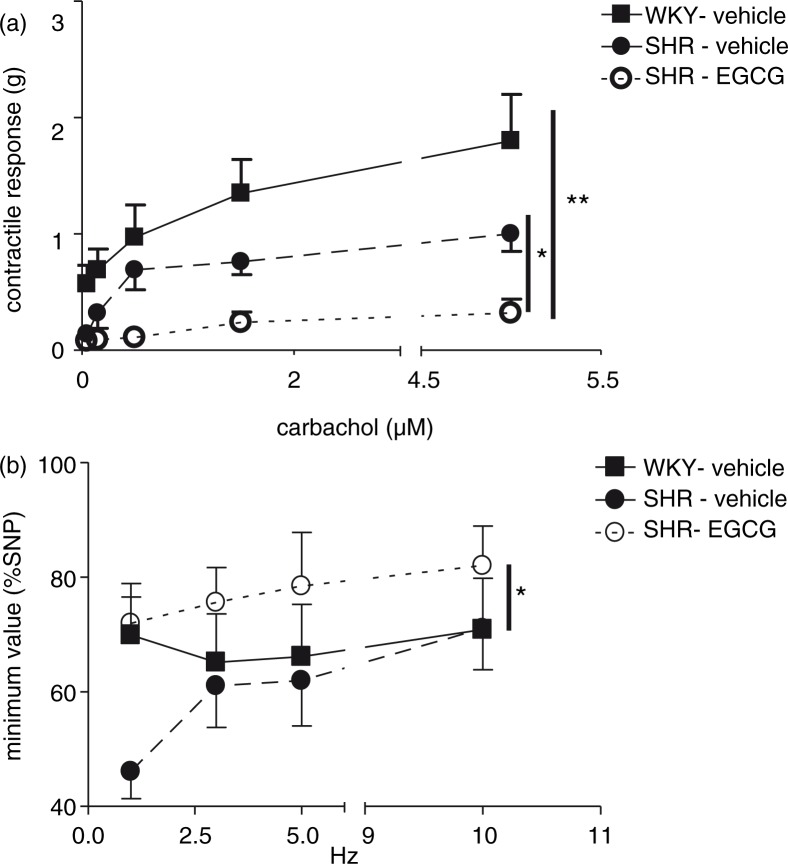
Effect of chronic EGCG treatment on *ex vivo* colonic and duodenal motility. (a) Colonic contractile responses to carbachol (0.05–5 µM); (b) duodenal inhibitory response to electrical field stimulation (EFS; 1–10 Hz, 13 V, 1 msec, 10-sec train duration) in preparation pretreated with N^G^-nitro-l-arginine (L-NNA, 20 min, 100 µM). Values are expressed as mean±SEM of 6–8 experiments. **p<0.01 vs vehicle-treated WKY rats; *p<0.05 vs vehicle-treated SHR.

Dose-dependent contractile responses to carbachol were not significantly different between specimens of colon obtained from vehicle-treated WKY rats and vehicle-treated-SHR. Interestingly, dose-response curve to carbachol was significantly reduced in colonic specimens from SHR exposed *in vivo* to chronic treatment with EGCG, when compared to either vehicle-treated WKY (**p<0.01) or vehicle-treated SHR (*p<0.05) (Fig. 6a).

Duodenal inhibitory response, measured as the minimum values of the basal tone reached during the relaxation to EFS (1–10 Hz), was not significantly different between vehicle-treated WKY rats and vehicle-treated-SHR. However, duodenum specimens from EGCG-treated SHR showed a significant increased response to L-NNA with respect to vehicle-treated SHR (*p<0.05), suggesting that EGCG treatment reduced the nitrergic component of the inhibitory response (Fig. 6b).

### Effect of *in vitro* acute EGCG incubation on duodenal and colonic motility

**Fig. 7:**
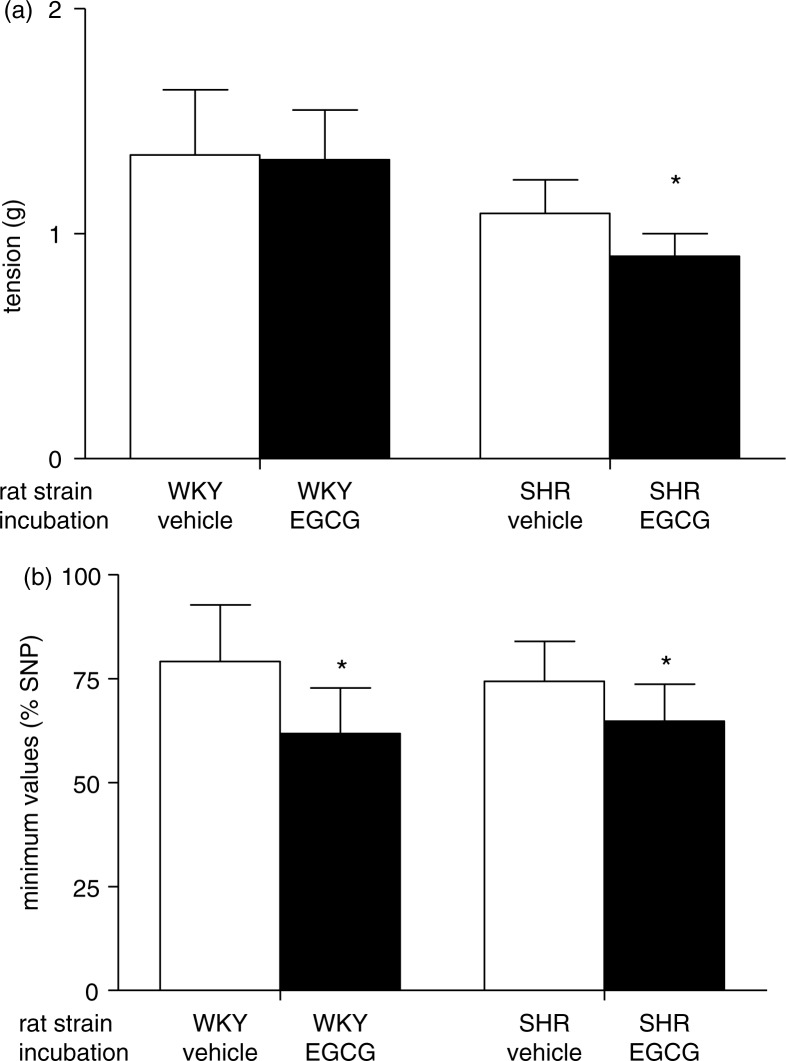
Effect of *in vitro* EGCG treatment on colonic and duodenal motility. (a) Colonic contractile responses to a fixed dose of carbachol (1.5 µM) in preparations from WKY rats and SHR after *in vitro* administration of either vehicle and EGCG (100 µM); (b) duodenal inhibitory response, expressed as minimum value reached by basal tone after a fixed frequency of electrical field stimulation (EFS; 5 Hz, 13 V, 1 msec, 10-sec train duration) in preparations from WKY rats and SHR after *in vitro* administration of either vehicle and EGCG (100 µM). Values are expressed as mean±SEM of 6–8 experiments. *p<0.05 vs its own vehicle.

To evaluate the effect of acute EGCG treatment, we examined the responses of colonic and duodenal specimens obtained from *in vivo* untreated WKY rats and SHR after 20 min *in vitro* incubation with EGCG (100 µM) or its vehicle (DMSO). Colonic contractile response to a fixed dose of carbachol (1.5 µM) was unaffected by *in vitro* acute exposure to EGCG in preparations from WKY rats, but it was significantly reduced in preparations from SHR when compared with respect to vehicle (*p<0.05) (Fig. 7a).

Duodenal specimens from both WKY rats and SHR showed a reduced inhibitory response, measured as the minimum values of the basal tone reached during the relaxation induced by a fixed EFS frequency (5 Hz, 13 V, 1 msec, 10-sec pulse train duration), when incubated with EGCG (100 µM) with respect to its own vehicle (*p<0.05) (Fig. 7b).

## Discussion

The present study shows that chronic treatment with EGCG for 3 weeks was able to prevent the weight gain in SHR and that this effect seems to be linked to the reduced food intake, in agreement with data previously obtained by our research group (14). Moreover, consistent with our previous investigation, we confirm that EGCG treatment restored both the reduced levels of adiponectin and insulin sensitivity in SHR as shown by the surrogate index QUICKI.

However, this study aimed to find out whether the lack of weight gain found in SHR chronically treated with EGCG could be due to: 1) EGCG effect on motor activity that, if increased, would consequently reduce the rate of body growth and/or 2) EGCG effect on duodenal and colonic motility (evaluated both *ex vivo* after chronic administration of EGCG and *in vitro* after acute incubation with EGCG) that may have affected food intake.

The present study shows that the lack of weight gain in EGCG-treated SHR cannot be attributed to the ability of chronic treatment with EGCG to increase motor activity. In fact, 3-week chronic administration of EGCG resulted in the opposite effect: the well-known hyperactivity of SHR (18, 19) that we confirmed in our study, was surprisingly reversed by EGCG. In particular, after 2 and 3 weeks of treatment with EGCG the horizontal activity (crossings) reached control levels observed in vehicle-treated WKY rats. The same effect was recorded in vertical activity (rearings) of SHR after 2 weeks administration of EGCG, although the beneficial effect disappeared after 3 weeks of treatment probably due to development of tolerance. When crossings and rearings were added and considered together, we observed an overall reduction of motor activity after 2 and 3 weeks of treatment in EGCG-treated SHR so that no difference could be detected with respect to vehicle-treated WKY rats. Our results suggest a central effect of chronic treatment with EGCG and, because SHR has been proposed as an animal model of attention-deficit/hyperactivity disorder (18–20), further studies may investigate the mechanisms of such effect even in order to extend the targets of this functional food.

Therefore, we had to leave out the hypothesis that the lack of weight gain found in EGCG-treated SHR could be attributed to the increased motor activity. However, we cannot rule out that since chronic treatment with EGCG was able to reduce the hyperactivity of SHR to the levels of vehicle-treated WKY rats, EGCG-treated SHR reduced their calories need so that they reduced food intake which resulted in the lack of weight gain.

Furthermore, we investigated the hypothesis that administration of EGCG could have effects at gastrointestinal level where it reaches high concentrations when ingested. The altered gastrointestinal motility could, in turn, cause after-effects such as reduced amount of nutrients absorbed and/or induction of early satiety that could result in the reduced food intake and lack of weight gain.

In this regard, several epidemiological studies have reported that increased consumption of green tea leads to reduced body fat and waist circumference, therefore showing an antiobesity effect (21–23). Those effects have been attributed to both reduced nutrient absorption and appetite inhibition either in man and rat (23). Moreover, literature data agree that alteration of intestinal motility can affect absorption of nutrients in the small intestine, suggesting that gastrointestinal motility could be a new target to treat obesity (24). Here we demonstrate that duodenal inhibitory response to EFS was significantly affected both after *in vivo* chronic treatment and after *in vitro* acute incubation with EGCG. In particular, duodenal specimens from SHR treated *in vivo* with EGCG showed an altered inhibitory response to EFS in the presence of L-NNA with respect to either vehicle-treated WKY rats or vehicle-treated SHR. Moreover, a decreased inhibitory response to EFS was recorded after *in vitro* incubation of duodenal preparations with EGCG in SHR, but also in WKY rats, which can be seen as a side effect of this functional food. Furthermore, chronic *in vivo* treatment and acute *in vitro* administration of EGCG reduced colonic contractile response to a cholinergic agent, carbachol, in SHR. Although the colon is the section of the gut less involved in absorption of nutrients, its motility may affect upper gastrointestinal motility with after-effects on satiety control and absorption of nutrients at the level of small intestine (25). Moreover, it has been reported that gastrointestinal parasympathetic inhibition is able to reduce food intake and body weight (26). Therefore, we may conclude that an altered gastrointestinal motility could contribute to the lack of weight gain seen in SHR treated with EGCG. We can also suggest that the reported beneficial effect of EGCG on weight gain (27, 28) may also be due to some local effect of this catechin.

Because pathologies related to increased body weight and obesity are becoming a major world problem, any functional food that could help to reduce body weight gain is very welcome, as it would be more accepted than any drugs used for the control of body weight. EGCG, as major catechin of green tea, is a good candidate to occupy a relevant position in this scenario.
